# Exploring the usefulness of real-time digitally supported fatigue monitoring in fatigue management: Perspectives from occupational therapists and brain injury survivors

**DOI:** 10.1177/03080226241269247

**Published:** 2024-09-10

**Authors:** Leisle Ezekiel, Harriet Wilding, Jeremy Dearling, Johnny Collett, Helen Dawes

**Affiliations:** 1 School of Health Sciences, University of Southampton, Southampton, UK; 2 School of Health Sciences, University of Southampton and Solent NHS Trust, Southampton, UK; 3 PPIE Representative, Oxford, UK; 4 Oxford Brookes University, Oxford, UK; 5 College of Medicine, NIHR Exeter, Exeter BRC, Exeter University, Exeter, UK

**Keywords:** Brain injury, fatigue, ecological momentary assessment, occupational therapy, self-management, digital technology

## Abstract

**Introduction:**

Persistent fatigue after acquired brain injury (ABI) needs long-term self-management. Self-monitoring supports self-management and informs the use of fatigue management strategies. Using ecological momentary assessment to monitor fatigue offers a data-driven approach to managing fatigue.

**Aims:**

To explore the usefulness of self-monitoring fatigue in real-time, using ecological momentary assessment to support self-management, from the perspective of people with ABI and occupational therapists.

**Methods:**

People with ABI monitoried their fatigue by wearing a Fitbit and completing six surveys a day on their phone for 6 days. Think aloud and semi-structured interviews elicited views on self-monitoring and the data generated. Transcripts were analysed using reflexive thematic analysis.

**Results:**

Four themes were developed from people with ABI (*n* = 9): (1) Attending to experience, (2) making sense of data, (3) the relationship between fatigue and activity, (4) implications for daily life. Three themes from occupational therapists (*n* = 5): (1) Challenges of using of data, (2) perceived benefits of self-monitoring, (3) viewing data in relation to their understanding of fatigue.

**Conclusion:**

Data generated in real-time challenged perspectives on fatigue and fatigue management. These insights may help people with ABI and their clinicians to plan personalised strategies for fatigue management and evaluate its impact on daily living.

## Introduction

Fatigue after acquired brain injury is a persistent problem with almost half of brain injury survivors experiencing disruptive fatigue six months post-injury, and around a third still experiencing fatigue two or more years later (Andelic et al., 2021; Elf et al., 2016). Fatigue is associated with a wide range of physical, cognitive, emotional and communication difficulties and is a barrier to participation in more complex occupations such as socialising, work, leisure and physical activity (Jackson et al., 2018; Rutkowski et al., 2021; Vincent-Onabajo and Adamu, 2014). Fatigue is associated with low levels of health-related quality of life and depression and a recent study found people with traumatic brain injury had 60% greater mental health care use than those without brain injury (Kureshi et al., 2023). Furthermore, there is evidence of high levels of health care use for several years post-brain injury (Obembe et al., 2019). Successful fatigue management is important for both the individual and health services.

Fatigue is difficult to manage because of its fluctuating and disruptive nature (Eriksson et al., 2022; Ezekiel et al., 2020; Lenaert et al., 2020). Hence, it is important for people with acquired brain injury (ABI) to understand how their fatigue fluctuates and if there are any associations and contextual factors that impact on the severity in order for them to effectively self-manage their fatigue long term, alongside the other problems arising from ABI, so that they can participate in routines and activities that are important to them.

Self-monitoring is a key behaviour change strategy and an important feature of self-management programmes (Dineen-Griffin et al., 2019; Michie et al., 2015). As explained by the PRIME theory of motivation, self-monitoring in real time supports an individual’s cognitive processes, subsequent evaluation of their fatigue experiences and development of personal rules or plans that then direct fatigue management behaviours (West and Michie, 2020). In fatigue management interventions, this is achieved through a combination of structured self-monitoring (using paper diaries) and in discussion with clinicians, to guide on coping strategies and help reduce the impact of fatigue on daily life (Drummond et al., 2021; Kim et al., 2022). But there is scope for a more data-driven approach to understanding fatigue, using everyday digital technology (such as smartphones, activity watches and digital assistants) and ecological momentary assessment (EMA).

EMA involves surveys delivered throughout the day and may be supported with wearable devices (e.g. activity monitors and smart watches) to enable dynamic monitoring of symptoms and behaviours (Shiffman et al., 2008). One clear advantage of EMA over more traditional assessment methods is that it removes the effect of recall bias as data are collected on experiences as they happen, rather than relying on an individual’s ability to remember past events. EMA has traditionally been used as a research data collection method rather than a clinical tool, but the use of EMA within clinical settings is now possible with the availability of smartphones and wearable devices. EMA has been shown to be feasible for use with people with brain injury (Juengst et al., 2015) but to support self-management, it needs to provide appropriate and timely feedback to those using it (Mitchell et al., 2022).

There are several proposed benefits of using data generated in this way, including greater autonomy as patients take more control of their fatigue management and subsequent health care decisions, increased personalisation of their care and more effective care as clinical decisions are based on patients actual experiences, rather than their remembered experiences (Austin et al.,2020; Reading & Merrill, 2018). However, to inform the development of data driven assessments and interventions within clinical practice, the views and opinions of patients and therapists are needed to determine if and how to optimise the process of self-monitoring of fatigue and suggest how patient-generated data may help manage fatigue.

### Aim

This study investigated how people with ABI perceived self-monitoring fatigue in real time and their views on the usability and usefulness of their digital active- and passive-generated data in supporting fatigue self-management. We also explored how occupational therapists viewed summarised data generated by the participants as they tracked their fatigue and activity.

## Methods

We conducted a qualitative enquiry, using reflexive thematic analysis, into the usefulness and usability of fatigue self-monitoring data for people with brain injury and fatigue, and for occupational therapists who deliver fatigue management. Two people living with brain injury provided feedback on the design of the study, the data collection methods and the data analysis. The study received ethical approval from the University of Southampton faculty ethics committee (study no. 76333) and participants provided informed consent.

### Recruitment

Individuals with brain injury were recruited after they responded to adverts on social media and through a NIHR brain injury Meditech cooperative. People with brain injury were eligible to take part in the research if they experienced fatigue after brain injury, were aged 18 or over, give informed consent, used a smartphone and were able to communicate sufficiently in English to participate in an interview. We used purposive sampling and aimed to select a heterogeneous sample to reflect the diversity of the affected population (considering ethnicity, gender, age and brain injury type).

Occupational therapists were recruited through Royal Colllege of Occupational Therapist’s specialist section of neurological practice.

Occupational therapists were eligible to participate if they had experience of delivering fatigue management interventions for people with acquired brain injury, gave informed consent and communicated sufficiently in English to participate in an interview.

### Data collection

People with brain injury completed 6 days of fatigue and activity monitoring using a wrist worn Fitbit Charge 4 activity monitor and by answering up to six EMA surveys a day, on their mobile phone. Participants received email notifications of a survey at pre-determined times between 9 am and 9 pm, through an online platform (Activepoints at https://activepoints.co.uk/). Fitbit data were also collected through the Activepoints platform. All data were encrypted when securely transferred to or exported from Activepoints.

In the survey, participants rated their current fatigue and energy levels and identified the type of fatigue experienced. They identified the activity they were mostly engaged in for 10 minutes prior to receiving the notification and rated their enjoyment and perceived mental and physical effort related to the activity on a 11-point numeric rating scale. The Fitbit derived physical activity data were time spent in physical activity and the intensity of the activity (determined by measuring heart rate through the watch sensor and applying a proprietary algorithm to classify physical activity intensity as sedentary, light, moderate or high).

Fitbit data and survey data were summarised using descriptive analysis, for example using frequency counts and mean scores or plotting variables together in bar charts.

Two members of the research team (LE and HW, first and second authors) conducted online, semi-structured interviews with people with brain injury about their experiences of monitoring fatigue and used the think-aloud technique to gather their perspectives on the usefulness of the data summaries (Table 1). The interview schedule was reviewed by people with lived experience of brain injury prior to use. LE also conducted online semi-structured interviews with occupational therapists where they viewed pseudonymised data summaries of fatigue and activity and asked them think aloud as they viewed the data. Participants with brain injury were supported to set up the Fitbit and their smartphone by the researchers, which meant they had met the interviewer online before being interviewed. Two occupational therapist (OT) participants were known to A in a professional capacity outside of the research study. A is a lecturer in Occupational Therapy with a PhD in Healthcare, experienced in qualitative research; B is a registered occupational therapist who worked as a research assistant during the study.

**Table 1.** Interview questions.

Interview questions for people with ABI	You have been wearing the Fitbit and answering questions about your fatigue as part of this study. What has that been like for you? Did you notice anything new about your activity or fatigue as you were tracking it? If yes, what did you notice? Is there anything else currently that you think is contributing to your fatigue (aside from brain injury). Think-aloud questions: What are you thinking as you look at the image? Please tell me more about what you are thinking. Looking back on the experience of tracking your fatigue and seeing your summarised information, has anything changed in the way you think about your fatigue? How have things changed? Has this process affected how you try to manage your fatigue in any way? If so, how? If not, why is that? How useful is this information to you in managing your fatigue? What would help to make this information more useful for you? Is there anything else you think would improve the information or improve the process of tracking your fatigue?
Interview questions for OTs	How long have you been practicing as an occupational therapist? What is your experience of working with people with brain injury and fatigue? Think-aloud questions: What are you thinking as you look at the image? (Probes -tell me more about what you are thinking, can you tell me about. .?). What information do you connect together as you look at the summaries? How useful would information like this be to you when working with people who have brain injury and fatigue? Please tell more about why it would or wouldn’t be useful? What would help to make this information more useful for you? What do you think the challenges are of using this information in clinical practice? What would make the information easier to understand? Is there anything else you think would improve the information? Is there anything that you think is missing from the information?

ABI: acquired brain injury.

#### Qualitative data analysis

Interviews were transcribed and analysed by A and B using reflexive thematic analysis (Braun and Clarke, 2022). We took a realist perspective during the analysis as we aimed to understand participants’ views on self-monitoring fatigue (Braun and Clarke, 2022). We also recognised that our assumptions and values around digital interventions would likely affect how we analysed the data. Hence, there was a need to use an approach where the researcher reflexivity was integral to the analytical process.

Interviews were conducted and recorded on MS Teams, the transcript downloaded, checked against the video of the interview for accuracy and imported into NVIVO for analysis. This process supported researchers to familiarise themselves with the data. Researchers noted their initial ideas and reflections not only on each interview but also on the data set as a whole. Transcripts from interviews with people with brain injury were analysed separately to those with occupational therapists.

Codes were refined throughout the coding process as researchers moved back and forth between interview transcripts. Our analysis was informed by beliefs around the connection between how people experience ‘doing’ their daily activities and health and well-being (Moll et al., 2015). After coding was completed, researchers developed initial themes and explored different thematic structures using thematic mapping. Once the final themes were developed, both researchers revisited the data and codes to check the validity of the themes.

## Results

Between November 2022 and May 2023, 16 people expressed interest and 15 people took part in the study: ten people with acquired brain injury and five occupational therapists (Table 2 for details). One person with brain injury withdrew from the study as they became unwell and struggled with setting up their smartphone; one person did not attend the interview. Recruitment ended because of the project timeframe. The mean percentage of surveys completed during the monitoring period was 78% (standard deviation of 20) with a range of 55 to 100%. Interviews lasted between 40 and 85 minutes.

**Table 2.** Description of participants.

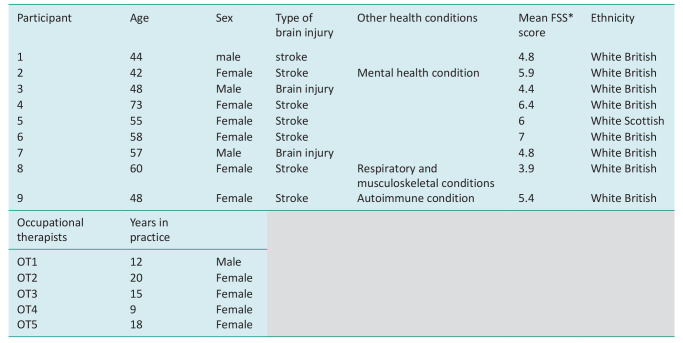

* FSS is the fatigue severity scale; VASF is visual analogue scale fatigue.

**Figure 1.**
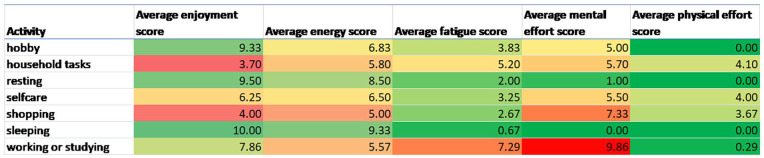
Heat map of activity and average numeric rating scale score out of 10 (with 10 being high) for fatigue, mental effort and physical effort. NB Green represents positive scores (e.g. less fatigue, less effort, more energy and enjoyment), red presents higher fatigue, higher effort, less enjoyment and less energy).

**Figure 2.**
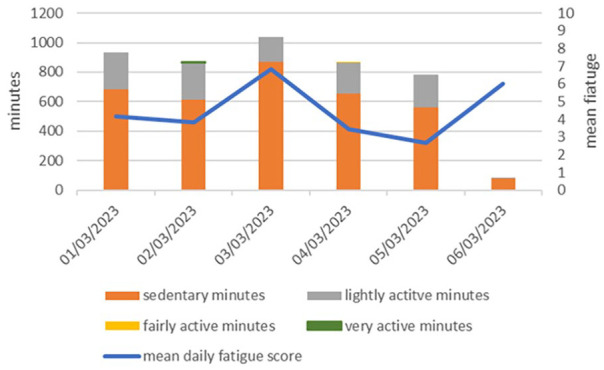
Daily mean fatigue score and sum of minutes spent in different intensities of physical activity across 24 hours. Sedentary minutes includes time spent asleep.

Please see Figures 1 and 2 for examples of the descriptive visual summaries of the survey data and Fitbit data that were created for each participant with brain injury. Please see Supplemental File for further examples of data visualisations.

### Themes

#### Thematic analysis of ABI interviews

**Figure 3.**
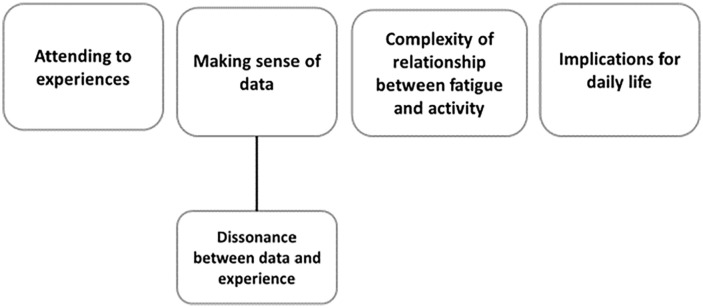
Map of final themes.

We developed four themes and one subtheme (Figure 3).

##### Theme 1: Attending to experiences

Most found the process of responding to surveys easy to do and were motivated to find out more about their fatigue. They found the process interesting and could have tracked for longer. Attending to their experiences in the moment not only was new for some but was also at odds with how they approached fatigue and they questioned whether paying closer attention to their fatigue was beneficial.

FSID 5: I’ve never really thought about things in this scale before. I just go through each day as it comes kind of thing, I think, especially since my stroke and the fatigues hit and what have you. I don’t try and second guess anything anymore. (Female, aged 55, stroke)

Participants described how completing the surveys raised awareness of what they were doing, the impact of their activities on their fatigue or highlighted how sedentary they were. Self-monitoring brought these things to their attention, but there was little information that was completely new to the participants.

FSID3: I think it made me more aware that when things are not going straight, it has an impact on my wellbeing more than anything. How I feel, how happy I am, how I cope, how I would go forward. And in the evenings, I was sitting on the sofa watching TV and like wow, there’s not much left. (Male, aged 48, TBI).

Few participants reported using the Fitbit app or looking at the watch to keep track of their physical activity, even if they usually wore a Fitbit. Three participants reviewed their sleep data on the Fitbit on occasion as they were curious about how the amount and quality of their sleep. One participant felt the Fitbit wasn’t relevant to her because she had difficulty walking, another participant was concerned about his privacy.

FSID7: Never done anything like that before because you can monitor everything, where to go and everything like that. And that’s just not me at all. (Male, aged 57, TBI)

##### Theme 2: Making sense of the information

Participants first tried to explain what they saw in the data by linking it to their memories of events or relating it to their general beliefs and understanding of their experience.

FSID 3: Obviously you can see, the highest is the mental fatigue. I agree with that because as I said, I sit there and work, and have to use my brain to engage with work. (Male, 48, TBI)

For some viewing, their summarised data validated their experience. Participant 6 discussed feeling very alone with her fatigue as those around her didn’t understand fatigue. But seeing her experience displayed in such a concrete way was empowering.

FSID6: But it was it was good in the way that it validated how I felt it, you know? It’s like, this is me, this is how it is and I feel like this and that’s OK to feel like this, cause you’ve got to live with it and you’ve got to just try and get the best out of it. (Female, 58, stroke)

Others saw their data and were please at how much they achieved.

FSID8 That’s really good actually. it just shows that I, I’m pretty active like two thirds of the time and resting a third, which is probably a good balance given who I am. (Female, 60, stroke)

However, several participants struggled to process the information or couldn’t remember what they had been doing during the self-monitoring period. Others saw relationships between their activity and fatigue but make assumptions about the direction of the relationship that can’t be confirmed by the data. For example, participant 5 notices:

*So the more I am sitting around, the higher the fatigue score. . .But more active I am the less it is* and goes on to explain why physical activity isn’t always tiring. She doesn’t consider the alternative explanations, such as choosing to do physical activities when her fatigue is low. (Female, 55, stroke)

### Sub-theme – Dissonance between the data and their recall of experience

Participants questioned the reliability of the data, whether they completed the survey correctly and the accuracy of the Fitbit when their summarised data didn’t match their experiences. Two participants questioned whether they made mistakes when answering the survey questions. Others reported that their fatigue was different to what they expected, or that they had more energy, more enjoyment than they expected.

FSID2: But look, I had more energy than I thought I have. My fatigue level is definitely high, as high as I thought they are. Actually, I think that’s probably higher than I thought they are. (Women, 42, stroke)

FSID 4: And those ( fatigue scores) are quite different on different days, which I’m quite surprised about because it feels mostly the same every (Female, 73, stroke).

#### Theme 3: Implications for daily life

Participants identified ways in which their data summary might influence their activities or their routines. For example, setting goals around fitness and physical activity or changing their exercise routine. One participant wanted to show her information to family members and health care professionals, to evidence her experiences.

FSID 6: And I really want to show my physio and probably my doctor as well, because you know, I talk so much about my fatigue to the doctor and my husband. (Female, 58, stroke).

Others expressed hesitancy at making changes in response to self-monitoring or viewing their data. Participant 3 when asked if summarised data would change his approach to managing his fatigue responded:

FSID3: I think it would do, but it’s something you have to work with because otherwise. . .what I’ve done over the last eight years, it’s what I do. But if you take on a new strategy then it takes time to work with that strategy. (Male, 48, TBI)

#### Theme 4: Complexity of relationships between activity and fatigue

The context of activity was key to understanding whether it triggered fatigue and the type of fatigue triggered. Participants explained how where the activity occurs, who with, what order of events, sometimes the combination of events or how people felt about the activity meant that a named activity could be fatiguing or not fatiguing.

FSID5: if physical exercise was walking your dogs. That would fatigue me, not because I’m walking, but because you’re concentrating on walking. You’re concentrating on what the dogs are doing and what have you. It’s all that thinking that goes into it. It’s not just going out for a walk for exercise. (Female, 55, stroke).

One participant talked about feeling fatigue and energised at the same time because he enjoyed the activity, even though it was challenging.

FSID1: fatigue and energy are separate because you’ve got the energy of, like, right, today we’re doing the school talk. I was tired of doing it ‘cause I am trying to control them, so that’s the fatigue. But I’ve got the energy because it’s giving me the boost in endorphins to do it. (Male 44, stroke)

### Themes from occupational therapist interviews

We developed three themes from our analysis of the occupational therapists’ interviews. These were: challenges of using self-monitoring data, interpreting the data through their fatigue ‘lens’, perceived benefits of self-monitoring fatigue.

*Challenges of using self-monitoring data*: OTs recognised that having the knowledge, time and resources to understand and interpret intensive self-monitoring and physical activity data were a potential challenge. They saw this type of data collection as being very different to approaches used in clinical practice. They also highlighted potential IT barriers within the NHS, such as challenges with access to software and computers.

OT3: this gives you quite a lot of rich data and then and it’s then as a therapist, you have to be pretty supple to be able to sort of help them interpret it.

OT4: So people need to be trained up and work out how to use it, how to interpret it properly. Actually if you’ve got more data than just a paper diary that’s obviously good in some ways. But at the time I think your team may value it further down the line but in that initial stage, it might be difficult.

Therapists also discussed how many of the sequelae of brain injury would affect service users ability to self-monitor and make use of their summarised data, even with support of an OT. These included cognitive, language, visual problems, fatigue and age, as well as their usual level of engaging with technology. Therapists perceive challenges for service users ‘turning their subjective experience in to quantitative data’ and questioned whether people understand scales sufficiently, are they rating what we think they are rating? One therapist was concerned about the impact of frequently interrupting people as they went about their day.

OT1 Uh, well, without any qualitative measure or subjective thing, it’s hard to turn experience into a quantity and to turn what is experience into a number, and that requires a certain degree of cognition for clients that we work with, particularly brain injury.

At times therapist did not perceive patterns in the data, and so the data were not seen as useful. At times the data summaries also contradicted therapists understanding of fatigue and so therapists questioned the reliability or validity of the data. Key areas of contradiction centred around self-ratings of energy and fatigue (where participants had scored energy as high whilst scoring their fatigue as high), or where participants described experiencing mental fatigue whilst engaging in physical activity.

*Perceived benefits of self-monitoring data summaries*. Most of the therapists described how the data would be useful to support conversations with patients, to facilitate better understanding of the context of daily activities, how activity linked to fatigue and the relationships between energy, enjoyment and effort. This information was potentially to support the choice of and pacing of activities. Therapists also thought it could a providing a baseline and help monitor change over time.

OT3: but then I’ve also got patients that are really on it and would really benefit from things like this because then then it starts to you can see the penny dropping with them when they’re given a little bit more rich richness to the data. Really, it’s dead easy for me to just say x + y equals z type thing, but they want to understand it a little bit for themselves.

Using charts to summarise and present different factors alongside each other was consistently thought to be useful as it provided a visual summary to support therapist and patient conversations. Therapists also valued the focus on occupations and that the data supported a strengths-based approach, that is, the data were not just about fatigue. One therapist explained how they try to do this using paper fatigue diaries, but thought this data provided a clearer visual summary.

OT1: It’s quite nice to sort of see that, what occupations even if they are difficult ones such as, you know, travelling potentially or working, that they actually enjoy it. . . . They enjoy work quite as much as other things, but they really enjoy exercising.

*Interpreting data in relation* to therapists understanding of fatigue. Therapists had differing perspectives of fatigue, and this meant their expectations of the data differed. Two therapists used mindfulness-based approaches in their fatigue interventions and so wanted to know about participant’s mindful engagement in their reported activities. Others asked their service users to report stress levels or other bodily symptoms of fatigue, to better understand the context of activity, rest and fatigue.

OT2: because if you think about the task negative network, you tend to ruminate over your problems and you’re worries, whereas it’s something like mindfulness or something that’s active resting.

OT4: not just if it’s physical or umm mental, but actually . . . do they have any other signs of fatigue? So have they started to develop a headache, or have they started a bit of word finding difficulty?

## Discussion

This study provides initial data supporting the potential benefits of self-monitoring to enhance self-management of fatigue in people with brain injury. Self-monitoring provided data that challenged participant’s recollection of how fatigue affects daily life, supported their ability to explain the impact of fatigue and highlighted the types of activities most likely to exacerbate their fatigue. Our findings are consistent with fatigue being a complex issue that requires a personalised approach to management (Lenaert et al., 2020), thus giving the data generated on an individual’s experience of fatigue utility. However, we also identified challenges in presenting and interpreting the data and barriers to clinical use which would need to be resolved before testing effectiveness and implementation.

In line with other studies, our participants found the experience of self-monitoring their subjective experiences and behaviour in real-time easy to do and were curious about using technology to monitor their fatigue (Juengst et al., 2015; Portanova et al., 2021). The process of answering questions about fatigue and activity as it happened drew participants’ attention to their experience and potentially increased their awareness of fatigue and activity. However, it should be considered that one person found attending to fatigue unhelpful, as not-attending to fatigue was used as a coping strategy. Attending to fatigue and managing activities in relation to fatigue are an established aspect of fatigue interventions for people with brain injury (Drummond et al., 2021; Ymer et al., 2021). This highlights the need to balance patients’ choice of approach with therapists’ perspectives, recognising that choice of coping strategy is value-laden.

Changes to attentional processes are common following brain injury and may be accompanied by limited self-awareness of deficits (El Husseini et al., 2023; Rabinowitz and Levin, 2014). Hence, attending in the moment to experiences of fatigue and activity may increase awareness of factors that trigger and exacerbate fatigue and support interventions such as activity pacing. Nevertheless, it is important to note that self-monitoring of fatigue in real time may lead to avoidance and withdrawal, rather than increasing engagement as increased focus on fatigue and potential drivers of fatigue may negatively affect mood states. Hence, it is vital for people with brain injury to have clinical support when initially self-monitoring and interpreting generated data.

The process of self-monitoring and using visual summaries of fatigue and activity experiences were perceived to be useful in empowering people to self-manage their fatigue. It is noteworthy that the overall experience of fatigue was largely driven by mental rather than physical effort. This information may to useful in supporting exercise and physical activity interventions. Indeed, therapists viewed increasing a patient’s awareness of their fatigue as beneficial and thought they might use such data to raise awareness of fatigue triggers and ineffective coping strategies. This is in line with findings from studies exploring clinicians’ approaches to fatigue management post-stroke (Thomas et al., 2019). Similar to Thomas et al., we also found that therapists’ clinical reasoning (as triggered by the data summaries) was shaped by their understanding of fatigue and this affected their interpretation of the data as well as views on what was missing from the data summaries. In our study, the therapists interpretation of data sometimes differed from those of participants with brain injury, particularly around approaches to pacing and relationships between physical activity and mental fatigue. This highlights the necessity for data to be trustworthy and for the clinician to understand the limitations of the data as they draw conclusions (Codella et al., 2018).

Whilst trustworthiness of the data is affected by data quality, we observed that differences between the situation as experienced first-hand and an individual’s recall of their experiences also caused participants to doubt the self-monitoring process and generated data. The discrepancy between real-time reporting and recalling events may be explained by the theory of temporal immediacy where real-time reporting reflects the ‘ground-truth’ of peoples’ experiences, and reflecting back is more of a narrative commentary (Cook et al., 2018). This reflecting back may be further hampered by cognitive challenges experienced by many people with for example changes in attention, information processing and memory (ABI). Temporal immediacy suggests that the experiencing self (how the individual feels in the moment) is more closely linked to behaviour change than the remembering self. Building self-awareness through attending to experiences in the moment and integrating such awareness into fatigue narratives may help to optimise the selection and use of fatigue management strategies (Cook et al., 2018). However, for this to happen, it is essential that the data collected is accurate and perceived to be reliable and trustworthy.

Our OT participants suggested several barriers to using self-monitoring and patient-generated data in clinical practice. It was seen as a new way of thinking about data and assessment which required advanced data literacy skills. Whilst they saw clear benefits, they also saw the time and resources needed to adopt such an approach as a major barrier to implementation. In addition, the benefits and cost-effectiveness of using self-monitoring and patient-generated data to support fatigue management have not yet been established. There is evidence that smartphone apps and wearables improve self-management across a range of health conditions, but further research is needed to understand their impact on managing fatigue (Rowland et al., 2020).

Despite UK policy imperatives to integrate digital technology into health and social care, the use of patient-generated data in routine practice continues to be hindered by poor integration with current health care systems, concerns about the quality of generated data and challenges in managing and interpreting large amounts of data (Abdolkhani et al., 2019; Health Education England, 2019; Lordon et al., 2020). Whilst the necessary digital literacy and digital capabilities of UK occupational therapists have now been defined and a range of strategies proposed to enable digitalisation of health and social care, little is known about how UK occupational therapists are integrating the therapeutic use of digital tools into routine practice (NHS England, 2019; RCOT, n.d.). Tack ( 2020) survey of allied health professionals (AHPs) use of digital technology revealed considerable variability in perceived digital competencies. Most of the respondents rated their competency to use and evaluate longitudinal patient data as fair to very poor and infrequently analysed such data in their practice. However, only 11% of 279 responses were completed by occupational therapists (Tack, 2020). Further research is needed to understand the factors affecting the therapeutic use of digital technology within occupational therapy practice.

### Limitations

Digital data collection methods affected the study in several ways. First, one participant decided to withdraw from the study because of technical difficulties. Sharing data summaries online for the interviews also meant that several participants struggled to see their data. This led to researchers deviating at times from the think-aloud interview process so as to orientate participants with the data. Whilst interviewers were mindful of maintaining a neutral position with regards to the data, it may be that participants views were influenced to a greater extent than if we had adhered strictly to the think-aloud process.

None of our participants was from an ethnic minority group. They were also several years post-injury and had established coping strategies, which may have affected our findings and the timing of such self-management interventions should be considered in future work. These limitations taken together affect the transferability of our findings to those from ethnic minority groups, those with more recent brain injury and those who struggle to use digital technology.

Our sample of occupational therapists was small and consisted of more senior practitioners with considerable expertise in fatigue management and so provide limited insight into the experiences of therapists using digital technology to support fatigue management.

### Implications

The needs of people with brain injury, and those supporting their care, must be central to the development and implementation of digital tools, particularly if used to support self-management.

Everyday technology is readily available and capable of capturing real-time data about users’ experiences. However, healthcare professionals need to have sufficient data literacy skills to understand the limitations of data collection and how to interpret the data generated.

Understanding stakeholder views is essential to address challenges experienced by occupational therapists in developing their digital capabilities and using digital tools to support their practice.

Whilst this study points to the potential usefulness of monitoring fatigue and activity in real time to support fatigue management, the benefits (in terms of efficacy and cost-effectiveness) still need to be established.

## Conclusion

This study revealed potential benefits of self-monitoring fatigue in real time using every day digital technology. Such an approach may be used to increase awareness of fatigue and to examine coping strategies. However, the complex nature of brain injury-related fatigue and consequences of brain injury mean that the likely scope of using this approach in fatigue self-management, without sufficient support, is limited. The study also highlights that managing and interpreting patient-generated data require health professionals to feel confident in their own and their patients’ data literacy skills. Further research is planned to investigate whether self-monitoring of fatigue in real time is feasible as an adjunct to fatigue management programmes.

**Key findings**

- Digital technology enables low burden, self-monitoring of fatigue in real-time.
- Interpreting patient-generated data requires high levels of data literacy.
- Self-monitoring and patient generated data may empower fatigue self- management.

**What the study has added**

This study provides unique insight into the benefits and challenges of using digital technology to monitor fatigue in real time, for people with brain injury and for occupational therapists.

## Supplemental Material

sj-docx-1-bjo-10.1177_03080226241269247 – Supplemental material for Exploring the usefulness of real-time digitally supported fatigue monitoring in fatigue management: Perspectives from occupational therapists and brain injury survivors Supplemental material, sj-docx-1-bjo-10.1177_03080226241269247 for Exploring the usefulness of real-time digitally supported fatigue monitoring in fatigue management: Perspectives from occupational therapists and brain injury survivors by Leisle Ezekiel, Harriet Wilding, Jeremy Dearling, Johnny Collett and Helen Dawes in British Journal of Occupational Therapy
