# Supplementary material for: Exploring the usefulness of real-time digitally supported fatigue monitoring in fatigue management: Perspectives from occupational therapists and brain injury survivors
Source: Br J Occup Ther. 2024 Sep 10;87(12):783–92. doi: 10.1177/03080226241269247 (PMC12033706; doi:10.1177/03080226241269247)
Supplement: sj-docx-1-bjo-10.1177_03080226241269247 – Supplemental material for Exploring the usefulness of real-time digitally supported fatigue monitoring in fatigue management: Perspectives from occupational therapists and brain injury survivors [file sj-docx-1-bjo-10.1177_03080226241269247.docx]

**Example visualisations of summarised data from self-monitoring fatigue and use of Fitbit .**

For the purpose of open access, the author has applied a Creative Commons attribution license (CC BY) to any Author Accepted Manuscript version arising from this submission.


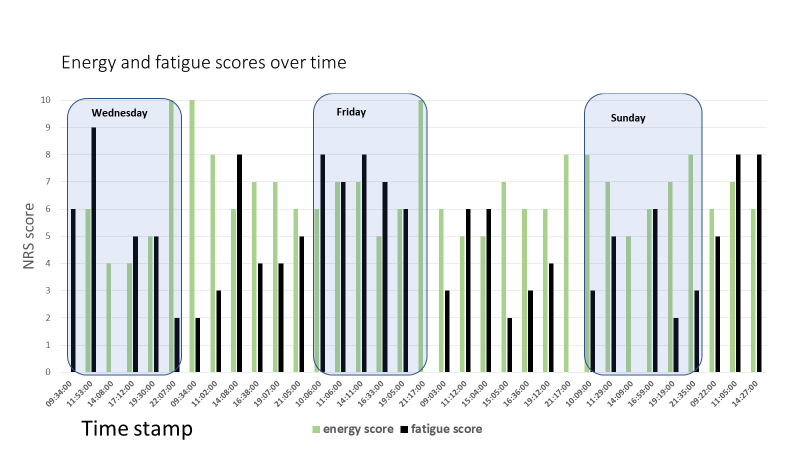


NRS fatigue : 0 no fatigue, 10 worst fatigue.


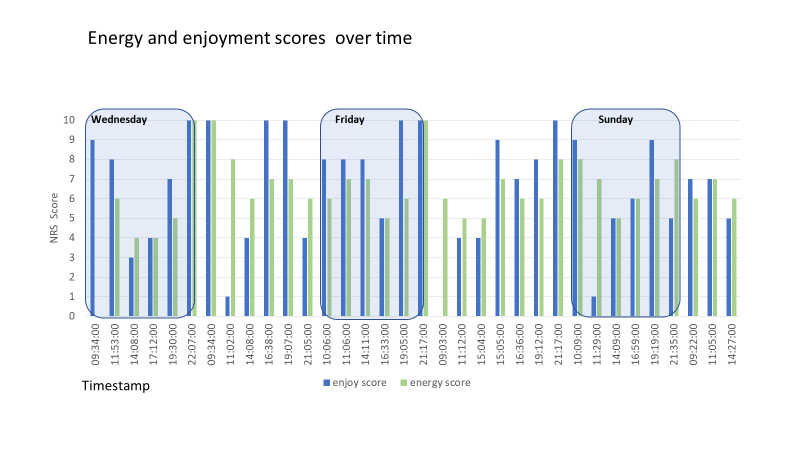


NRS energy: 0 no energy, 10 full of energy. NRS enjoyment: 0 not at all enjoyable, 10 very enjoyable.

Participant id: FSid7


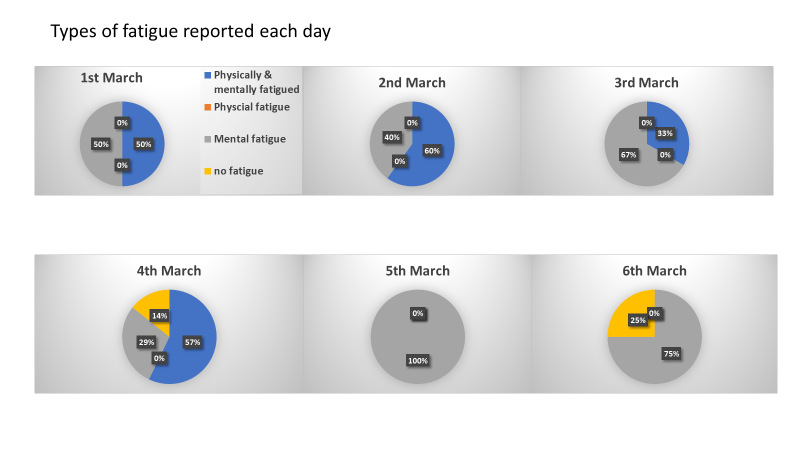


Participant FSid3


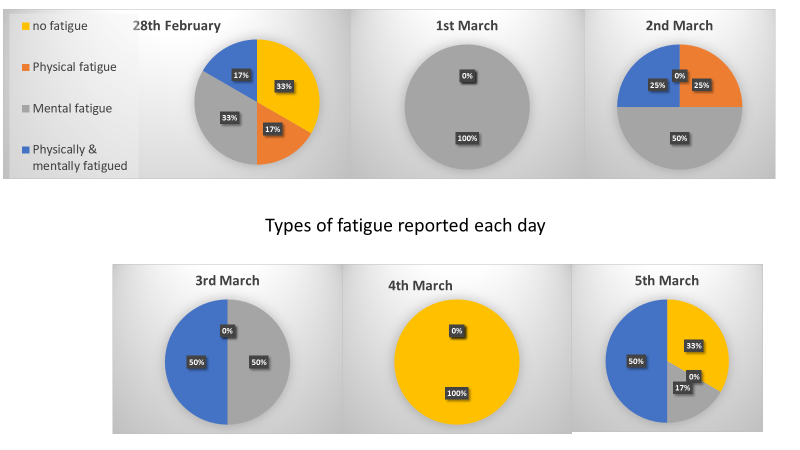


**Participant FSID7**


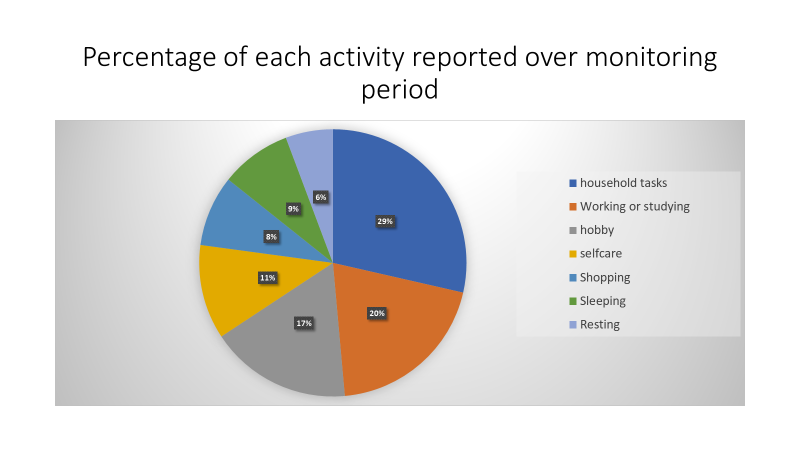


Participant FSID3


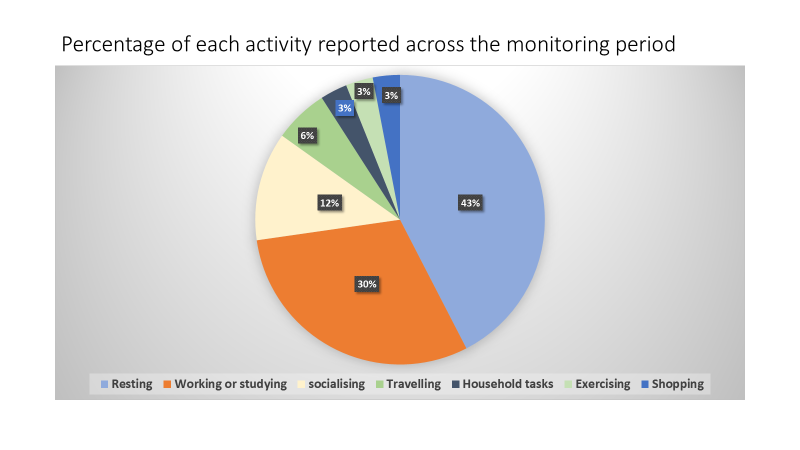


Participant FSID2: heat map of activities with mean energy, enjoyment, effort and fatigue scores.

Participant FSID5


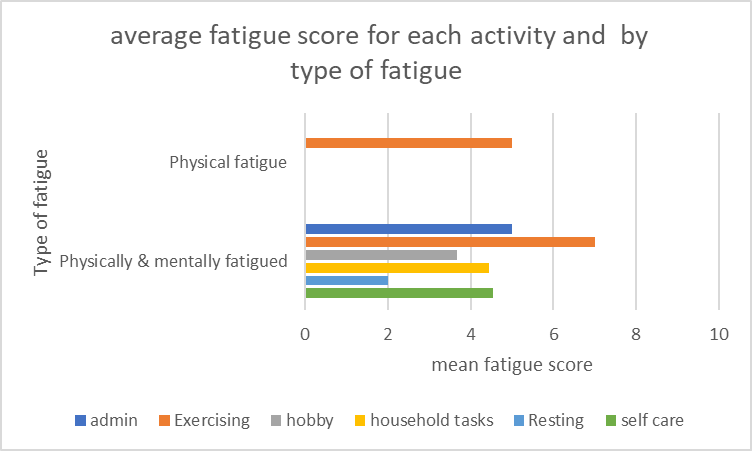


Participant FISD3


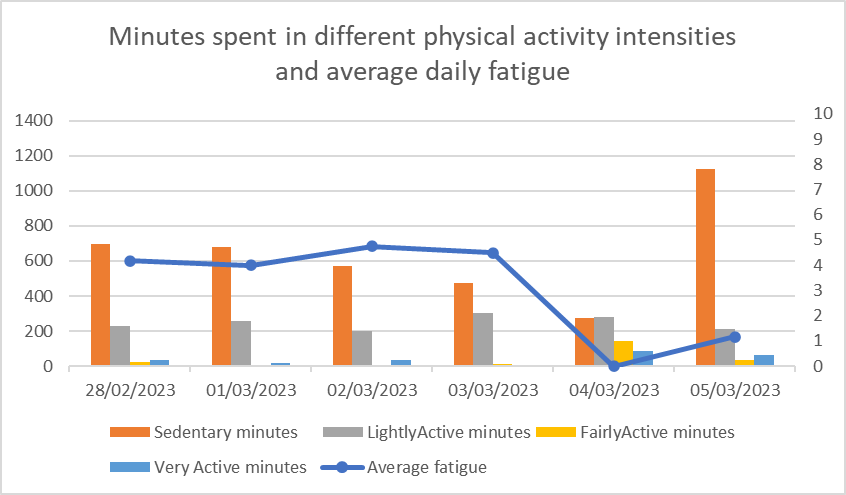


NB: sedentary minutes include time resting or asleep.
